# Characterisation of cold-induced mitochondrial fission in porcine aortic endothelial cells

**DOI:** 10.1186/s10020-021-00430-z

**Published:** 2022-01-31

**Authors:** Leonard Quiring, Björn Walter, Niklas Lohaus, Dhanusha Schwan, Anja Rech, Andrea Dlugos, Ursula Rauen

**Affiliations:** 1 Klinische Forschergruppe 117, Universitätsklinikum Essen, Essen, Germany; 2 Institut für Physiologische Chemie, Universitätsklinikum Essen, Hufelandstr. 55, 45147 Essen, Germany; 3 Present Address: Institute of Diagnostic and Interventional Radiology, University Hospital Zurich, Zurich, Switzerland; 4 Present Address: Department Hamm 2, Hochschule Hamm-Lippstadt, Hamm, Germany

**Keywords:** Mitochondrial dynamics, Mitochondrial fragmentation, Mitochondrial fusion, Endothelium, Mitochondria, Preservation

## Abstract

**Background:**

Previously, we observed that hypothermia, widely used for organ preservation, elicits mitochondrial fission in different cell types. However, temperature dependence, mechanisms and consequences of this cold-induced mitochondrial fission are unknown. Therefore, we here study cold-induced mitochondrial fission in endothelial cells, a cell type generally displaying a high sensitivity to cold-induced injury.

**Methods:**

Porcine aortic endothelial cells were incubated at 4–25 °C in modified Krebs–Henseleit buffer (plus glucose to provide substrate and deferoxamine to prevent iron-dependent hypothermic injury).

**Results:**

Cold-induced mitochondrial fission occurred as early as after 3 h at 4 °C and at temperatures below 21 °C, and was more marked after longer cold incubation periods. It was accompanied by the formation of unusual mitochondrial morphologies such as donuts, blobs, and lassos. Under all conditions, re-fusion was observed after rewarming. Cellular ATP content dropped to 33% after 48 h incubation at 4 °C, recovering after rewarming. Drp1 protein levels showed no significant change during cold incubation, but increased phosphorylation at both phosphorylation sites, activating S616 and inactivating S637. Drp1 receptor protein levels were unchanged. Instead of increased mitochondrial accumulation of Drp1 decreased mitochondrial localization was observed during hypothermia. Moreover, the well-known Drp1 inhibitor Mdivi-1 showed only partial protection against cold-induced mitochondrial fission. The inner membrane fusion-mediating protein Opa1 showed a late shift from the long to the fusion-incompetent short isoform during prolonged cold incubation. Oma1 cleavage was not observed.

**Conclusions:**

Cold-induced mitochondrial fission appears to occur over almost the whole temperature range relevant for organ preservation. Unusual morphologies appear to be related to fission/auto-fusion. Fission appears to be associated with lower mitochondrial function/ATP decline, mechanistically unusual, and after cold incubation in physiological solutions reversible at 37 °C.

**Supplementary Information:**

The online version contains supplementary material available at 10.1186/s10020-021-00430-z.

## Introduction

Organs are stored under hypothermic conditions (in special preservation solutions) to preserve integrity and function for transplantation. While hypothermia has beneficial effects, delaying injury caused by ischemia and subsequent energy deficiency, it also leads to cold-induced injury (Belzer et al. 1988; Rauen et al. 1999, 2002). The major mechanism of cold-induced injury to diverse cell types has been shown to be an increase in the intracellular chelatable iron pool leading to the formation of highly reactive oxygen species and an iron-dependent induction of a mitochondrial permeability transition (Kerkweg et al. 2002; Rauen et al. 2000, 2003, 2004). As an additional change occurring in cells incubated under hypothermic conditions, we observed a pronounced mitochondrial fission after cold incubation of liver endothelial cells for 18 h at 4 °C in University of Wisconsin solution (Kerkweg et al. 2003). This fission occurred both in the absence and in the presence of the iron chelator deferoxamine. Subsequently, marked fission was observed under hypothermic conditions in many cell types relevant in transplantation medicine, such as hepatocytes (Pless et al. 2012; Rauen et al. 2003), corneal endothelial cells (Rauen et al. 2006), and kidney epithelial cells (Hendriks et al. 2017; Zhang et al. 2010)/renal tubules (Bienholz et al. 2017). In these studies, mitochondrial fission was observed after different periods of cold incubation (18 h–5 days) at 4 °C or 10 °C and in many different storage solutions.

In principle, mitochondrial fission is a physiological process. Under normothermic conditions mitochondria form a dynamic network in the cell by continuous fission and fusion, processes crucial for equal distribution of mitochondria to daughter cells (Taguchi et al. 2007), for quality control (Chan 2012; Twig et al. 2008) and for the response to varying energy requirements (Pagliarini et al. 2008), with the interconnected network being more efficient in ATP production (Pernas et al. 2016). The processes of these mitochondrial dynamics are highly regulated. Under normothermic conditions mitofusin (Mfn) 1 and 2 and optic atrophy protein 1 (Opa1) are responsible for fusion of the outer and inner mitochondrial membrane (Cipolat et al. 2004; Ishihara et al. 2004; Song et al. 2009), respectively, while dynamin-related protein 1 (Drp1)—in conjunction with its receptors fission 1 protein (Fis1), mitochondrial fission factor (Mff), mitochondrial dynamics proteins of 49 kDa (MiD49) and of 51 kDa (MiD51)—is regarded to be crucial for mitochondrial fission (Hoppins et al. 2007; Kraus et al. 2021; Otsuga et al. 1998, van der Bliek et al. 2013).

While the mechanisms of mitochondrial fission have been described in detail for normothermic conditions (Kraus et al. 2021), little is known about the mechanisms and consequences of cold-induced mitochondrial fission. Furthermore, its temperature dependence is unclear but of great interest with regard to evolving subnormothermic preservation methods (Kaths et al. 2018; Moers et al. 2009). Therefore, we here study the occurrence and mechanisms of cold-induced mitochondrial fission in endothelial cells, i.e. in a cell type known to be very sensitive to hypothermia (Bath et al. 2019; Nordling et al. 2018, Rauen et al. 2004), and under conditions where this mitochondrial alteration is not superimposed by mitochondrial permeability transition and/or cellular injury.

## Methods

### Isolation and culture of porcine aortic endothelial cells

Porcine aortae were obtained from the local slaughterhouse. Endothelial cells were isolated mechanically as described by Peters et al (Peters 2005). The cells were cultured in M199 cell culture medium (Biochrom, Germany) supplemented with fetal calf serum (20% *v/v*), L-glutamine (2 mM) and antibiotics (100 U/ml penicillin, 100 U/ml streptomycin) under a humidified 5% CO_2_ atmosphere and split 1:3 on day 4. Three days later the cells were again split 1:3 and used for experiments after 48 h in an early confluent state.

### Cold incubation/rewarming

For experiments the cells were washed with Hanks’ Balanced Salt Solution (HBSS, 37 °C) and incubated in modified Krebs–Henseleit (KH) buffer (143.6 mM Na^+^; 128.3 mM Cl^−^; 25.0 mM HCO_3_^−^; 5.9 mM K^+^; 1.2 mM Mg^2+^; 1.2 mM SO_4_^2−^; 1.2 mM H_2_PO_4_^−^; 2.5 mM Ca^2+^; 20.0 mM HEPES) supplemented with glucose (5 mM) and deferoxamine (1 mM, Novartis, Basel, Switzerland), the latter added to prevent iron-dependent cellular and mitochondrial injury (Rauen et al. 2004). The cold incubation solution was added at room temperature, and cells were kept under a 5% CO_2_, 21% O_2_ and 74% N_2_ atmosphere (for the maintenance of the bicarbonate buffer system) at 4 °C or at other temperatures specified in the results section (Rauen et al. 1999). For some experiments, the Drp1 inhibitor Mdivi-1 (20 µM, Enzo Life Sciences, Farmingdale, New York, USA) or DMSO as solvent control were added to the cells for a 15 min pre-incubation in KH buffer (+ 5 mM glucose) as well as to the cold incubation solution. For some experiments, the tubulin polymerization inhibitor nocodazole (5 µM, Sigma-Aldrich, St. Louis, Missouri, USA) or DMSO (solvent control) was added 18 h before and during rewarming (rewarming with 5% instead of 20% fetal calf serum). After different periods of cold incubation, cells were washed once with cold HBSS, cold cell culture medium was added and cells were slowly rewarmed at 37 °C. For some control conditions, cycloheximide (50 µg/ml; Applichem, Darmstadt, Germany), α-amanitin (10 µg/ml; Sigma-Aldrich, St. Louis, Missouri, USA) or carbonyl cyanide *m*-chlorophenyl hydrazone (CCCP; 20 µM; Sigma-Aldrich, St. Louis, Missouri, USA) were added to the incubation solution.

### Mitochondrial morphology

For microscopic studies cells were grown on collagen-coated coverslips. The cells were washed with HBSS and stained with MitoTracker Red CMXRos (150 nM; Invitrogen, Carlsbad, CA, USA) in KH buffer with glucose (5 mM) for 20 min at 37 °C. After washing the cells were incubated dye-free for 2 h in KH buffer with glucose and then exposed to cold conditions as described above.

The cells were fixated with PFA (3.7% *w/v*) in cell culture medium for 10 min at incubation temperature followed by 15 min at 37 °C. The slides were mounted on an object slide and analysed using fluorescence microscopy (Axio Observer.Z1 with Apotome1, Plan-Apochromat 63x/1.40 Oil DIC, Zeiss, Germany; λ_exc._ = 546 ± 6 nm, λ_em._ ≥ 590 nm).

For live cell imaging the cells were incubated on the microscope in the Cooling/Heating Incubation Insert P-Set 2000 (Pecon, Erbach, Germany) at the indicated temperature with 5% CO_2_ (maintained at the same temperature). To protect the cells from phototoxicity, excitation light intensity (Colibri.2, Zeiss, Germany; 2% intensity), the frequency of the exposures (every 20 min at 8 °C or every 5 min at 37 °C) and the exposure time (2000 ms) were reduced to a minimum.

### Immunofluorescence

For immunofluorescence MitoTracker-stained cells were fixated, residual PFA was quenched with NH_4_Cl (50 mM) in PBS for 30 min, cells were permeabilized with Triton X-100 (0.5% *v/v*) and, after washing, blocked according to the manufacturer’s instructions. The cells were covered with the antibody (Drp1: Cell Signalling, #8570, 1:50 dilution, 1 h at RT; α-Tubulin, Sigma Aldrich, St. Louis, Missouri, USA, T5168, 1:500, 1 h at RT), washed and incubated with the secondary antibody (Anti-Rabbit IgG-FITC, Sigma-Aldrich, #F9887, 1:100, 45 min at RT; anti-Mouse IgG-FITC, Sigma-Aldrich, F9137, 1:100, 1 h at RT), then mounted and analysed by fluorescence microscopy (λ_exc._ = 470 ± 20 nm, λ_em._ = 525 ± 25 nm).

### Mitochondrial membrane potential

Washed cells were counterstained with MitoTracker Green (500 nM; Molecular Probes, Eugene, Oregon, USA) in KH buffer with glucose (5 mM) for 20 min at 37 °C.

Then cells were stained with TMRM (500 nM; Molecular Probes, Eugene, Oregon, USA) for 20 min. During experiments, a 100 nM maintenance dose was added to the incubation solution. Cells were analysed by fluorescence microscopy (TMRM: λ_exc._ = 546 ± 6 nm, λ_em._ ≥ 590 nm; MitoTracker Green: λ_exc._ = 470 ± 20 nm, λ_em._ = 525 ± 25 nm).

### Electron microscopy

For electron microscopy cells were fixed using 5% *(w/v)* glutaraldehyde and 8% *(w/v)* paraformaldehyde in PHEM buffer (60 mM PIPES, 25 mM HEPES, 10 mM EGTA, 2 mM MgCl_2_, pH 7.2; Schliwa et al. 1981) for 30 min at incubation temperature followed by 30 min at room temperature. Afterwards cells were further fixed using 2.5% *(w/v)* glutaraldehyde and 4% *(w/v)* paraformaldehyde in PHEM buffer for 2 h at room temperature. Then cells were processed as described before (Polishchuk et al. 2013). Samples were cut in ultrathin sections (55 nm) using a Leica EM TRIM2 (Leica, Wetzlar, Germany). Sections were analysed using the Transmission Electron Microscope System JEOL-JEM 1400 Plus (JEOL Ltd., Tokio, Japan) of the central imaging facility of the Universitätsklinikum Essen (IMCES) at 120 kV.

### Cell viability

Hoechst 33342 and propidium iodide staining was performed and LDH release measured as previously described (Rauen et al. 1999). Incubation times for Hoechst 33342 were extended under cold conditions to 2 h.

### ATP measurement

The ATP content of cells grown on 6-well plates was determined using the ATP Bioluminescence Assay Kit CLSII (Roche, Mannheim, Germany) according to the manufacturer’s instructions.

### Western blot

Cell extract preparation was performed as described previously (Bienholz et al. 2017). The shown proteins were visualised using a rabbit antibody against Phospho-Drp1-S637 (Cell Signaling, Danvers, Massachusetts, USA, #4867), Phospho-Drp1-S616 (Cell Signaling, #4494), Drp1 (Cell Signaling, #5391), Opa1 (Cell Signaling, #80471), Fis1 (Thermo Scientific, Waltham, Massachusetts, USA, #PA5-22142), Mfn1 (Merck, Darmstadt, Germany, #ABC41), Caspase-3 (Cell Signaling, #9662), Mff (Cell Signaling, #84580), Phospho-Mff (Cell Signaling, #49281), MiD51 (Proteintech, Rosemont, Illinois, USA, 20164-1-AP), MiD49 (Proteintech, 28718-1-AP) and a mouse antibody against Oma1 (Santa Cruz, Dallas, Texas, USA, sc-515788) and Mfn2 (Abcam, Cambridge, United Kingdom, #ab56889), all in 1:1000 dilution. Anti-rabbit IgG (Sigma-Aldrich, St. Louis, Missouri, USA, #A8275) and anti-mouse IgG (Sigma-Aldrich, #A4416) conjugated with peroxidase was used as secondary antibody in 1:5000 dilution. The chemiluminescence signals (SuperSignal™ West Femto Maximum Sensitivity Substrate, Thermo Fisher Scientific, Waltham, Massachusetts, USA) were recorded using the gel documentation system Fusion Pulse 6 (Vilber Lourmat, Eberhardzell, Germany) and quantified by the software Bio1D (Vilber Lourmat, Eberhardzell, Germany). Gels stained with Coomassie were used as a loading control.

### Statistics

All experiments were performed in duplicate and repeated four times. Statistical analysis was performed with GraphPad Prism (GraphPad Software, San Diego, CA, USA). The Friedman test was used for all statistical calculations; p ≤ 0.05 was considered significant.

## Results

### Cold-induced mitochondrial fission

**Fig. 1:**
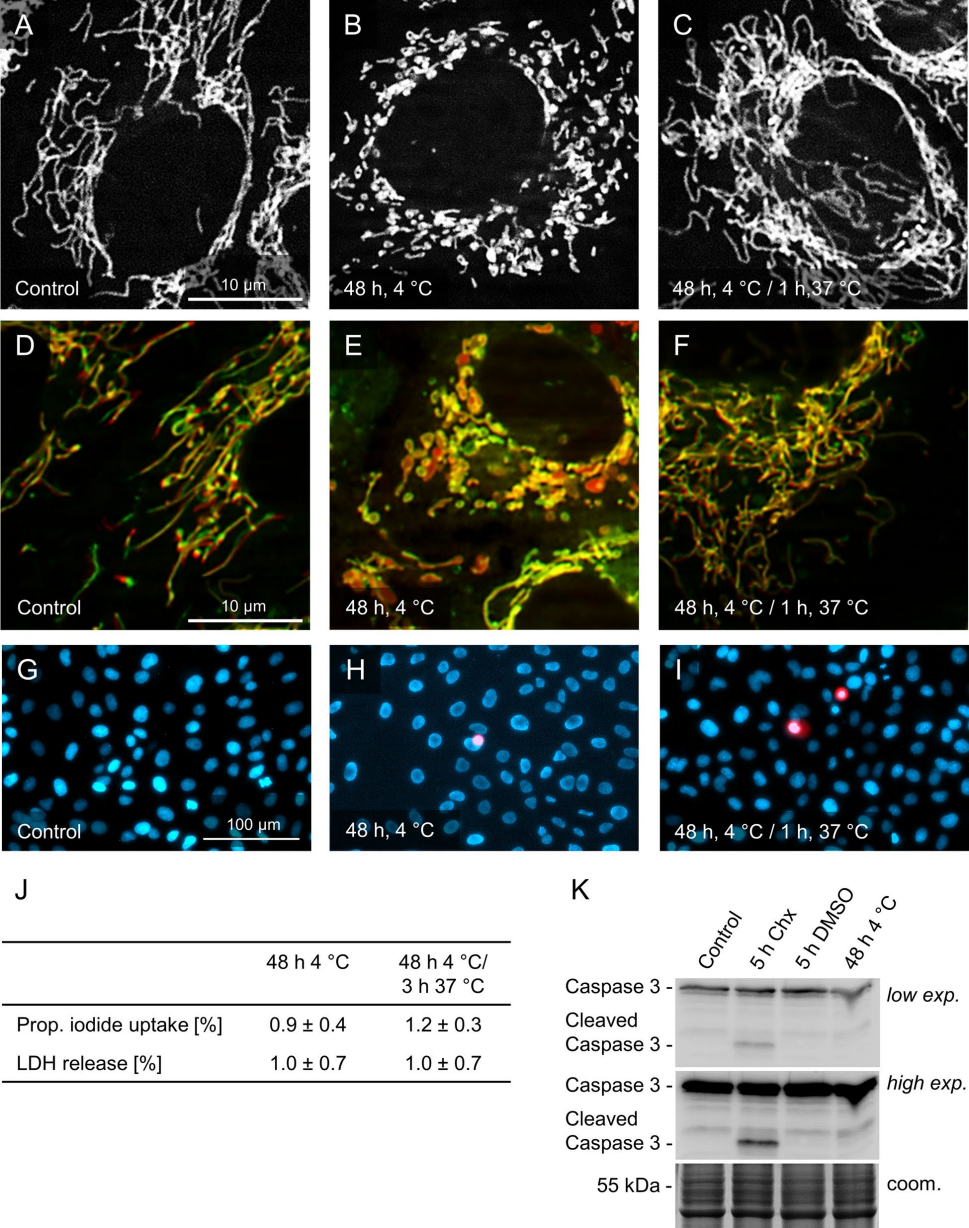
Cold-induced mitochondrial fission and its reversibility. Mitochondria of porcine aortic endothelial cells stained with MitoTracker Red (**A**–**C**) are shown before cold incubation (**A**), after cold incubation at 4 °C for 48 h (**B**) and after rewarming for 1 h at 37 °C (**C**). Mitochondrial membrane potential (TMRM staining, red) is shown before cold incubation (**D**), after cold incubation for 48 h (**E**) and after rewarming (**F**) in mitochondria counter-stained with MitoTracker Green (green). Cell viability was assessed by propidium iodide uptake and Hoechst 33342 counter-staining (**G**–**I**), its quantification (**J**) and assessment of LDH release (**J**). Caspase 3 was analysed as an apoptosis marker; cycloheximide (Chx; 50 µg/ml) served as positive control (**K**). Representative figures of n = 4 experiments; for **J** means ± SD of n = 4 experiments. (MitoTracker Red/TMRM/PI: λ_exc._ = 546 ± 6 nm, λ_em._ ≥ 590 nm; MitoTracker Green: λ_exc._ = 470 ± 20 nm, λ_em._ = 525 ± 25 nm; Hoechst: λ_exc._ = 359 ± 24 nm, λ_em._ = 445 ± 25 nm)

Under normothermic control conditions, porcine aortic endothelial cells stained with MitoTracker Red showed a network of long filamentous mitochondria (Fig. 1A). After 48 h of cold incubation (4 °C), however, mitochondria showed marked fission (Fig. 1B). This fission was reversible when the cells were rewarmed for 1 h at 37 °C (Fig. 1C).

### Mitochondrial membrane potential of fragmented mitochondria

Co-staining with MitoTracker Green (Fig. 1D–F; green) and tetramethylrhodamine (TMRM; red)—resulting predominantly in co-localization of fluorescence in the overlay—confirmed that mitochondria fragmented at 4 °C and demonstrated that the fragmented mitochondria in the cold still had mitochondrial membrane potential.

### Cell viability

Staining with Hoechst 33342 and propidium iodide showed that during cold incubation and after rewarming cell injury only affected very few cells (< 1.5%; Fig. 1G–J). Similar data were obtained assessing the release of lactate dehydrogenase (LDH; Fig. 1J). Caspase-3 cleavage, as marker of apoptosis, could not be observed after 48 h of cold incubation, although the positive control (cycloheximide; Alessenko et al. 1997) was positive (Fig. 1K)—confirming that cold-induced mitochondrial fission was not due to cell injury/apoptosis.

### Time dependence of mitochondrial fission

**Fig. 2:**
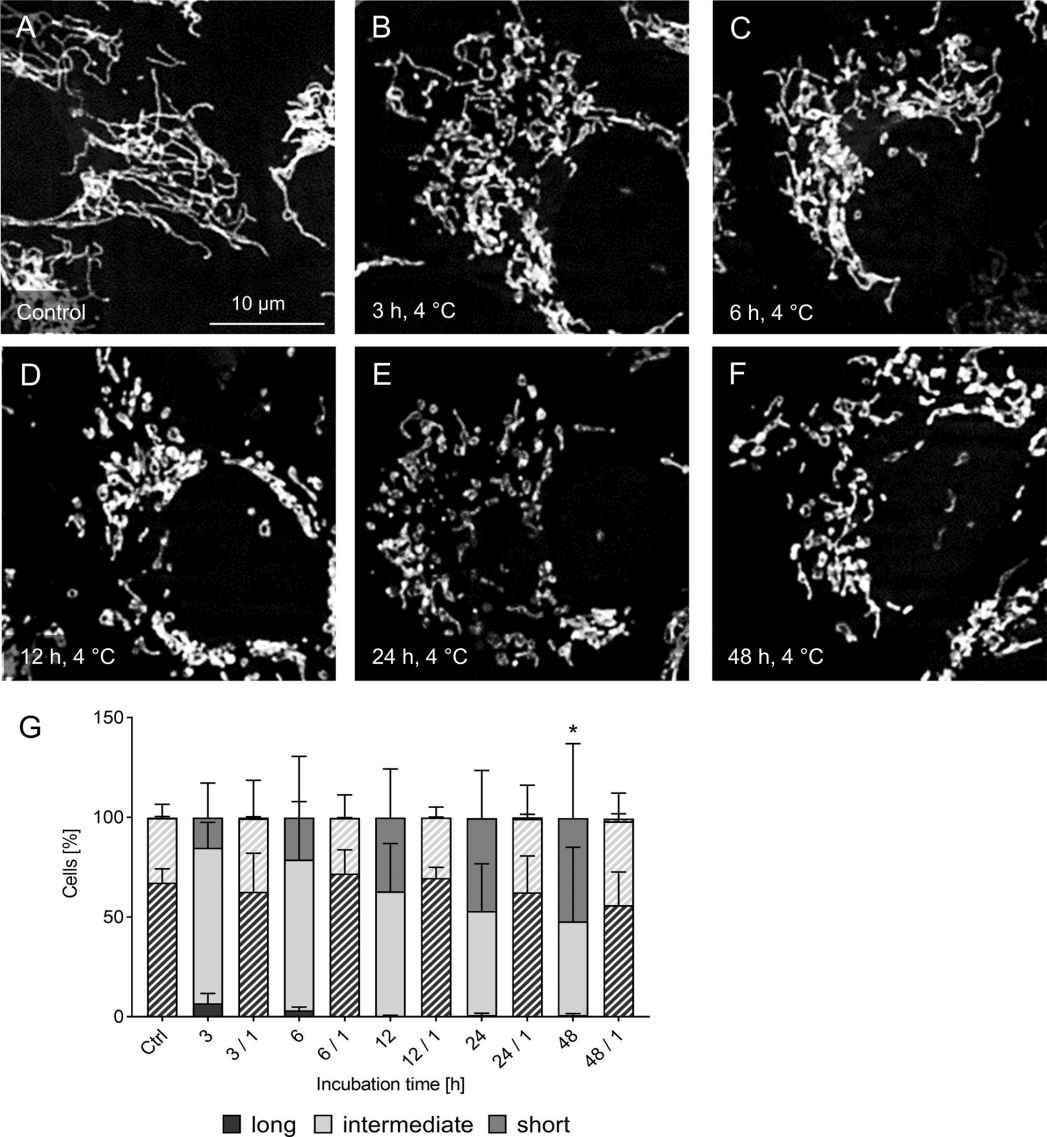
Time dependence of cold-induced mitochondrial fission. Cultured porcine aortic endothelial cells (**A**) were exposed to hypothermia for 3 h (**B**), 6 h (**C**), 12 h (**D**), 24 h (**E**) and 48 h (**F**). Mitochondria were stained by MitoTracker Red and documented using fluorescence microscopy (λ_exc._ = 546 ± 6 nm, λ_em._ ≥ 590 nm). Representative figures of n = 4 experiments. For quantification (**G**) cells, incubated at 4 °C for the indicated time (i.e. 3 h) and rewarmed for 1 h at 37 °C (e.g. 3/1), were categorised in cells with predominantly short, intermediate or long mitochondria by a person blinded to the experiment. Warm/rewarming conditions are shown hatched. Means ± SD of n = 4 experiments. *Significant difference of long mitochondria compared to control (p ≤ 0.05)

When cells were incubated for different time periods at 4 °C, mitochondria showed fission as early as after 3 h (Fig. 2): The long tubular mitochondria typical for this cell type (Fig. 2A) fragmented in the cold to yield predominantly intermediate length mitochondria, with only a few elongated mitochondria left (Fig. 2B, G). With ongoing cold incubation, cold-induced fission became more marked (Fig. 2C–G). After 6 h most mitochondria were of intermediate length with an increase of short mitochondria. After 12 h of hypothermia there were no long (tubular) mitochondria left. After 24 h and 48 h of cold incubation the number of intermediate mitochondria decreased and short mitochondria predominated after 48 h. After 1 h of rewarming at 37 °C, marked re-fusion could be observed after all cold incubation periods up to 48 h (Fig. 2G). The cold-induced mitochondrial fission progressing over time was visualized in a time-lapse video at 8 °C (the lowest temperature technically possible for prolonged live cell imaging) which is available as Additional file 1 in the supplementary data. The reversibility of cold-induced fission is also shown in a time-lapse video performed during rewarming after cold incubation for 48 h at 4 °C (Additional file 2).

### Temperature dependence of cold-induced mitochondrial fission

**Fig. 3:**
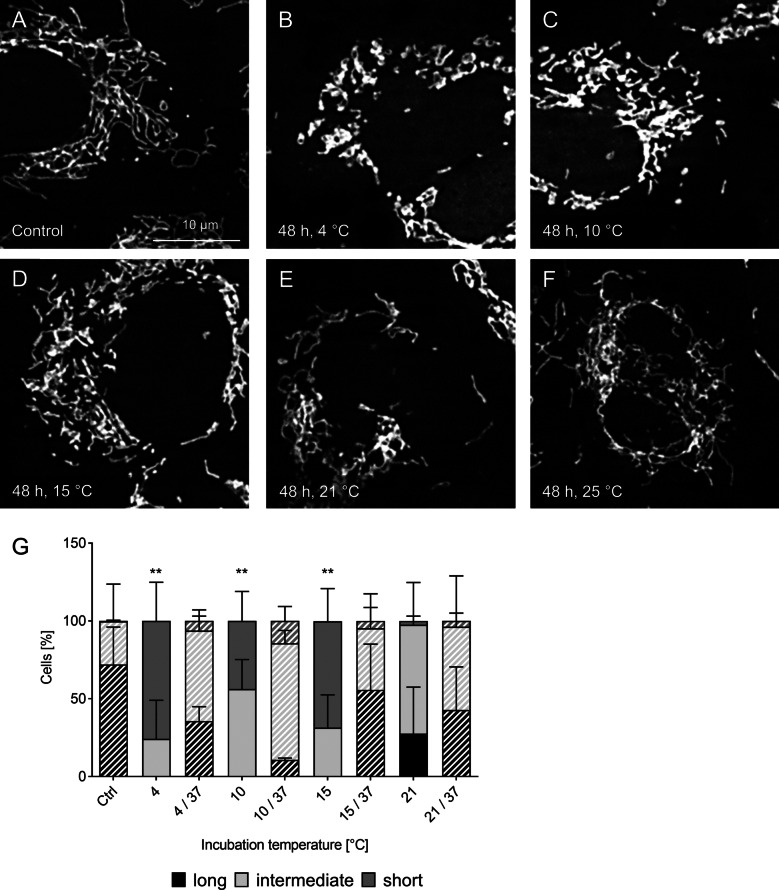
Temperature dependence of cold-induced mitochondrial fission. Porcine aortic endothelial cells (**A**) were incubated for 48 h at 4 °C (**B**), 10 °C (**C**), 15 °C (**D**), 21 °C (**E**) and 25 °C (**F**). MitoTracker Red-stained mitochondria were documented by fluorescence microscopy (λ_exc._ = 546 ± 6 nm, λ_em._ ≥ 590 nm). Representative figures of n = 4 experiments. For quantification (**G**) cells were categorised in cells with predominantly short, intermediate or long mitochondria by a person blinded to the experiment. Warm/rewarming conditions are shown hatched. Means ± SD of n = 4 experiments. **Significant difference of long mitochondria compared to control (p ≤ 0.01)

The extent of cold-induced mitochondrial fission showed a clear temperature dependence when cells were incubated for 48 h at temperatures ranging from 4 °C to 25 °C. At 4 °C the already described loss of long mitochondria and predominance of short mitochondria was noted (Fig. 3B). A similar result was observed at 10 °C and 15 °C with the disappearance of long mitochondria and a high percentage of short mitochondria (Fig. 3C, D). At 21 °C, in contrast, a large difference to the lower temperatures was visible, with only few short mitochondria, a predominance of intermediate length mitochondria and a considerable proportion of long mitochondria (Fig. 3E). At 25 °C the long morphology dominated with intermediate length mitochondria also being present (Fig. 3F). After “cold” incubation at all incubation temperatures the mitochondria had re-fused again after 1 h rewarming at 37 °C (Fig. 3G).

### Occurrence of unusual morphologies with longer cold incubation periods

**Fig. 4:**
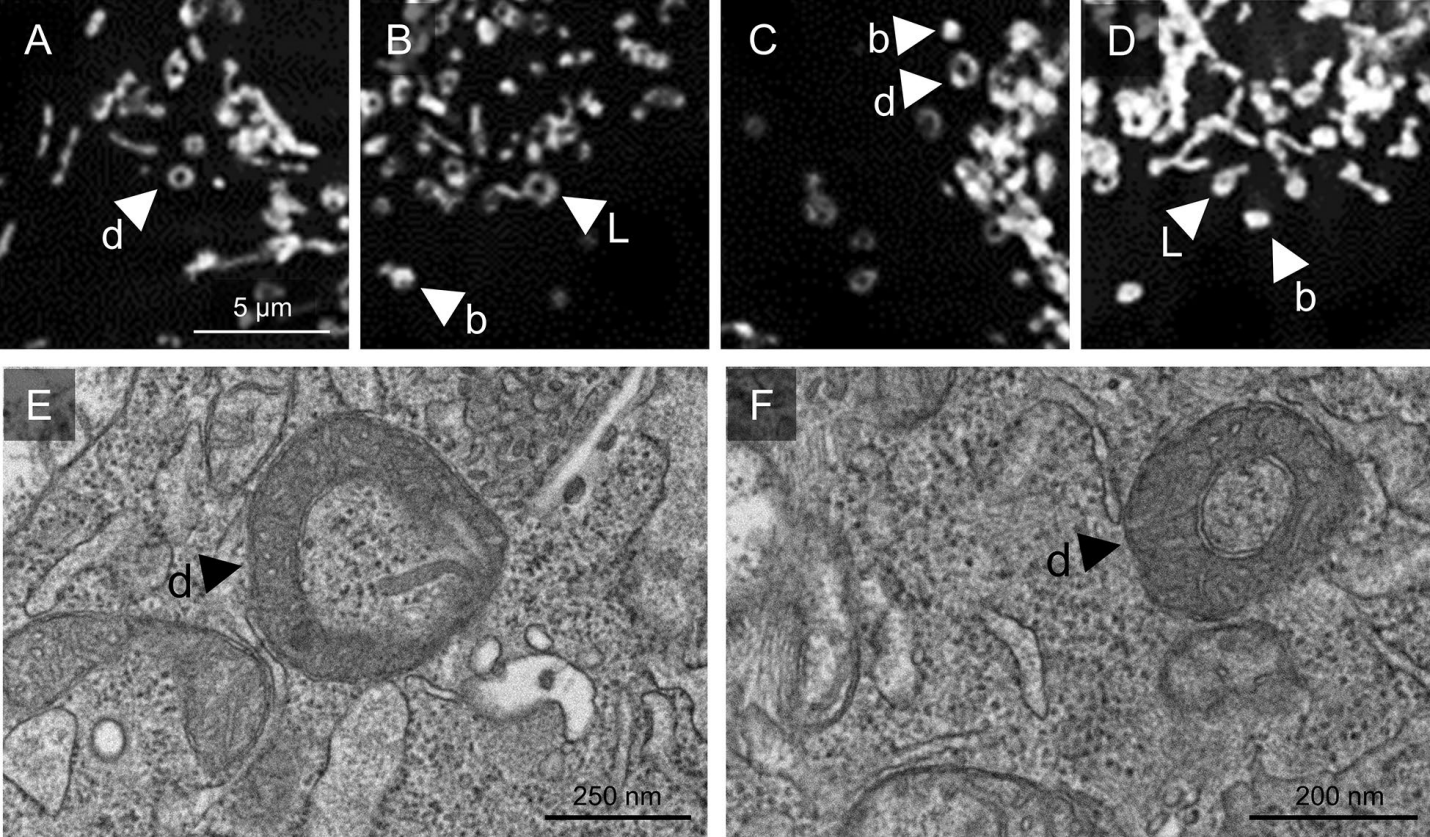
Unusual morphologies of mitochondria during cold incubation. Cells incubated at 4 °C for 48 h (**A**–**D**) showed unusual mitochondrial morphologies such as donut (d in **A**, **C**), blob (b in **B**–**D**) or lasso structures (L in **B**, **D**). The images were documented using fluorescence microscopy (λ_exc._ = 546 ± 6 nm, λ_em._ ≥ 590 nm; **A**–**D**). Electron microscopy (**E**, **F**) confirmed the occurrence of donut-like mitochondrial morphologies (d in **E**, **F**) already after 6 h of cold incubation

In addition to enhanced fission under hypothermic conditions mitochondria displayed further morphology changes (Fig. 4). Not only short or dot-like very short mitochondria but also donuts (ring-like mitochondria), blobs (swollen mitochondria/swollen donuts) and lasso-like mitochondria formation occurred in the majority of cells. Especially the typical donut morphology occurred in most cells under cold incubation (Fig. 4A) with blob and lasso morphologies present alongside it. These morphologies were observed after ≥ 6 h at 4 °C or after 48 h at ≤ 15 °C, increasing over time. In the ultrastructure a closed circular morphology of a proportion of the mitochondria (donut) could be observed (Fig. 4E, F).

### Influence of cellular growth state/confluence

**Fig. 5:**
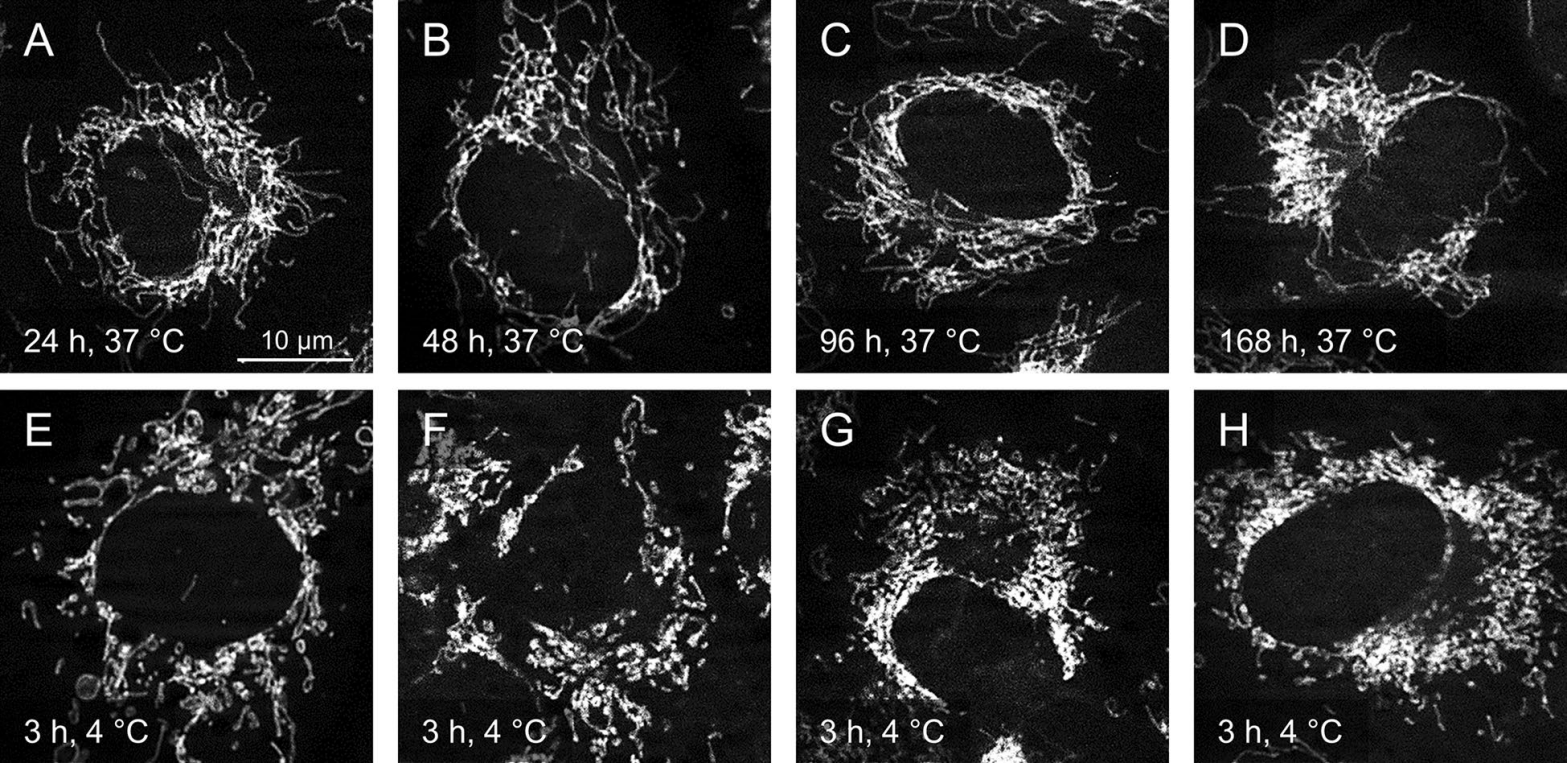
Growth dependence of cold-induced mitochondrial fission. Porcine aortic endothelial cells were cultured before cold incubation for 24 h, 48 h, 96 h and 168 h at 37 °C (**A**–**D**). Thereafter, the cells were incubated for 3 h at 4 °C (**E**–**H**). MitoTracker Red-stained mitochondria were documented by fluorescence microscopy (λ_exc._ = 546 ± 6 nm, λ_em._ ≥ 590 nm). Representative figures of n = 4 experiments

Because in a previous publication endothelial sensitivity towards cold storage conditions had been shown to be influenced by cellular growth state (Rauen et al. 1994b), with proliferating cells being less sensitive to cold storage conditions than late-confluent cells, a potentially similar dependence of cold-induced mitochondrial fission on cellular growth state/culture period was assessed here by modifying the usual 48 h culture period to 24 h, 96 h and 168 h. While under all culture conditions long filamentous mitochondria predominated before cold incubation (Fig. 5A–D), cold-induced mitochondrial fission was visible throughout all experiments (Fig. 5E–H). The longer the culture period, the more marked the fission appeared already after 3 h of cold incubation. In the late-confluent cells cultured for one week (168 h) the observed fission was most marked (Fig. 5H).

### Effects of cold incubation on Drp1 phosphorylation, Drp1 receptors and Drp1 localization

**Fig. 6:**
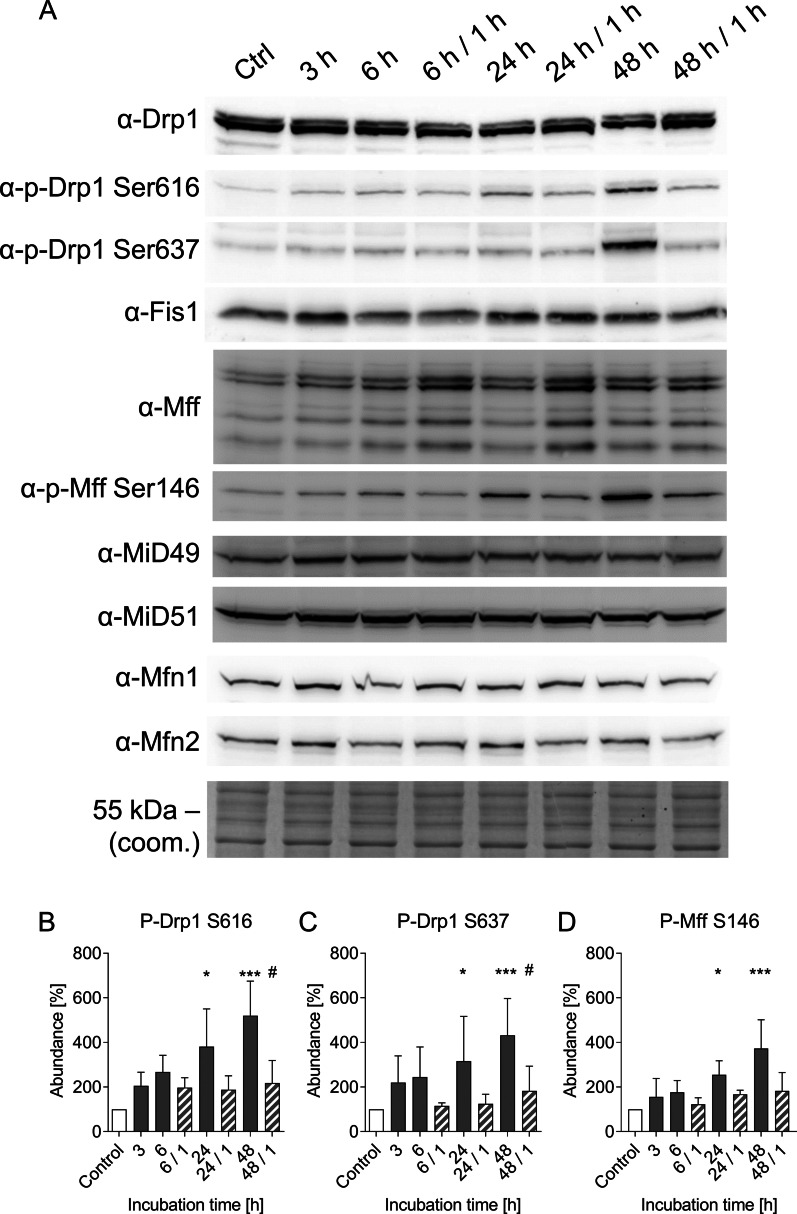
Qualitative and quantitative analysis of proteins involved in mitochondrial fission and outer membrane fusion. Protein lysates of porcine aortic endothelial cells incubated for 3 h, 6 h, 24 h or 48 h at 4 °C and rewarmed for 1 h at 37 °C were analysed by western blot (**A**) for protein levels of Drp1 and phosphorylation of Drp1 (activating site p-Drp1-S616 and inactivating site p-Drp1-S637) and Fis1. Additionally Mff, p-Mff Ser146, MiD49, MiD51, Mfn1 and Mfn2 were targeted. Coomassie staining was used as loading control (coom.). Representative figures of n = 4 experiments. The band intensity was quantified using the BioVision software and normalized to the control (**B**–**D**). Rewarming conditions are shown hatched. Means ± SD of n = 4 experiments. * Significantly different to control (p ≤ 0.05) *** (p ≤ 0.005) # Significantly different to the respective cold incubation (p ≤ 0.05)

Drp1, the key enzyme of the fission machinery, showed no distinct alteration in protein levels neither under hypothermia nor after rewarming (Fig. 6). The same applied to its receptors Fis1, which is also considered to have a regulatory role (Yu et al. 2020), Mff, MiD49 and MiD51 and the two key enzymes for fusion of the outer mitochondrial membrane Mfn1 and Mfn2 (Fig. 6). In contrast to the total protein levels the phosphorylated isoforms of both Drp1 and Mff showed an increase over the cold incubation period. However, both the activating site S616 and the inactivating site S637 of Drp1 were phosphorylated in the cold with significant changes after 24 h and 48 h (Fig. 6B and C). Phosphorylated Mff showed a similar time course (Fig. 6D). When cells were rewarmed (37 °C) after each cold incubation period, the level of phosphorylated Drp1 and Mff decreased within 1 h to a level largely matching the control before cold exposure.

**Fig. 7:**
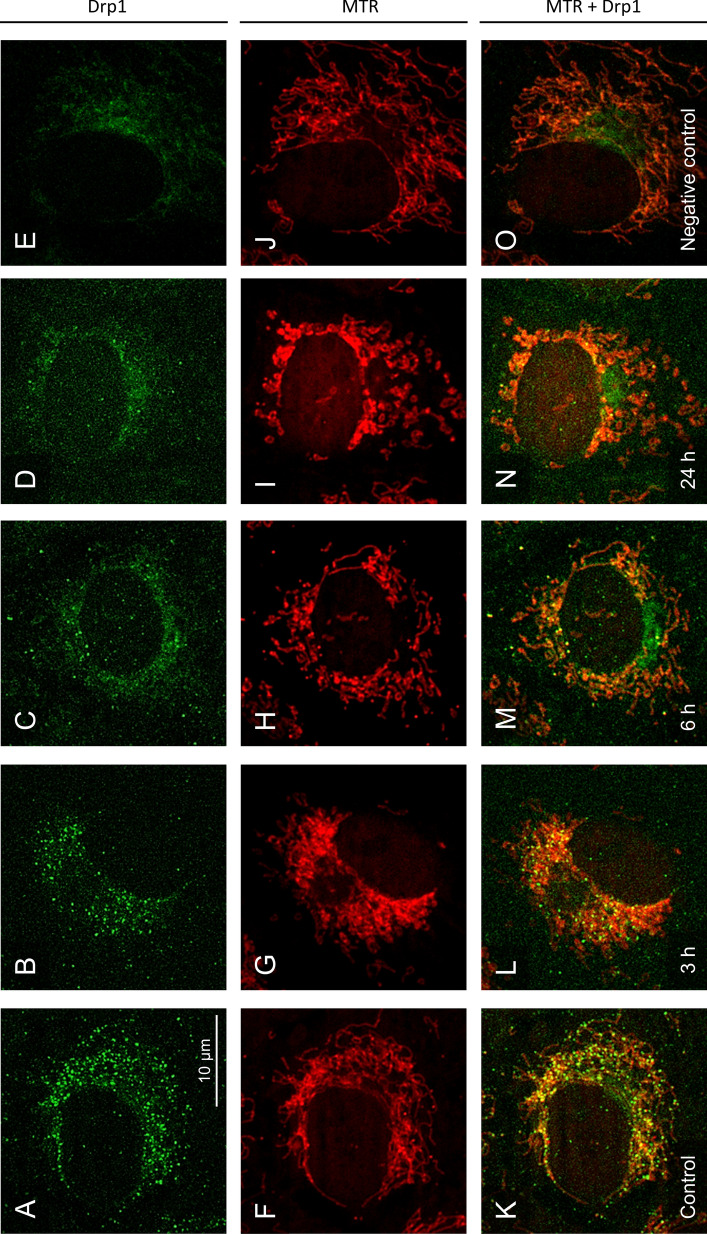
Immunofluorescene for Drp1 localisation under hypothermic conditions. Drp1 localization was assessed using a Drp1 antibody and a FITC-labelled secondary antibody (**A**–**E**, green) in porcine aortic endothelial cells that had been stained with MitoTracker Red to show mitochondria (**F**–**J**, red). Merged images show cells (control cells, **K**) after cold incubation for 3 h (**L**), 6 h (**M**) and 24 h (**N**) and a staining without Drp1 antibody (only secondary antibody) as negative control (**E**, **J**, **O**). The images were documented by fluorescence microscopy (MitoTracker Red: λ_exc._ = 546 ± 6 nm, λ_em._ ≥ 590 nm; FITC: λ_exc._ = 470 ± 20 nm, λ_em._ = 525 ± 25 nm). Representative figures of n = 4 experiments

As both the activating and the inactivating phosphorylation site of Drp1 were phosphorylated under hypothermic conditions, the localization of Drp1 was analysed by immunofluorescence. In control cells not exposed to hypothermia punctate Drp1 staining could be observed (Fig. 7A) which co-localized to mitochondria (Fig. 7K). After a cold treatment of 3 h to 6 h at 4 °C both the punctate staining and the co-localization with mitochondria decreased (Fig. 7L, M). After 24 h cold incubation—the time duration in which the cold-induced mitochondrial fission was practically completed (Figs. 2G, 7I)—almost no co-localization of the Drp1 antibody to mitochondria and mostly weak diffuse fluorescence in the cytosol (Fig. 7N) could be observed.

**Fig. 8:**
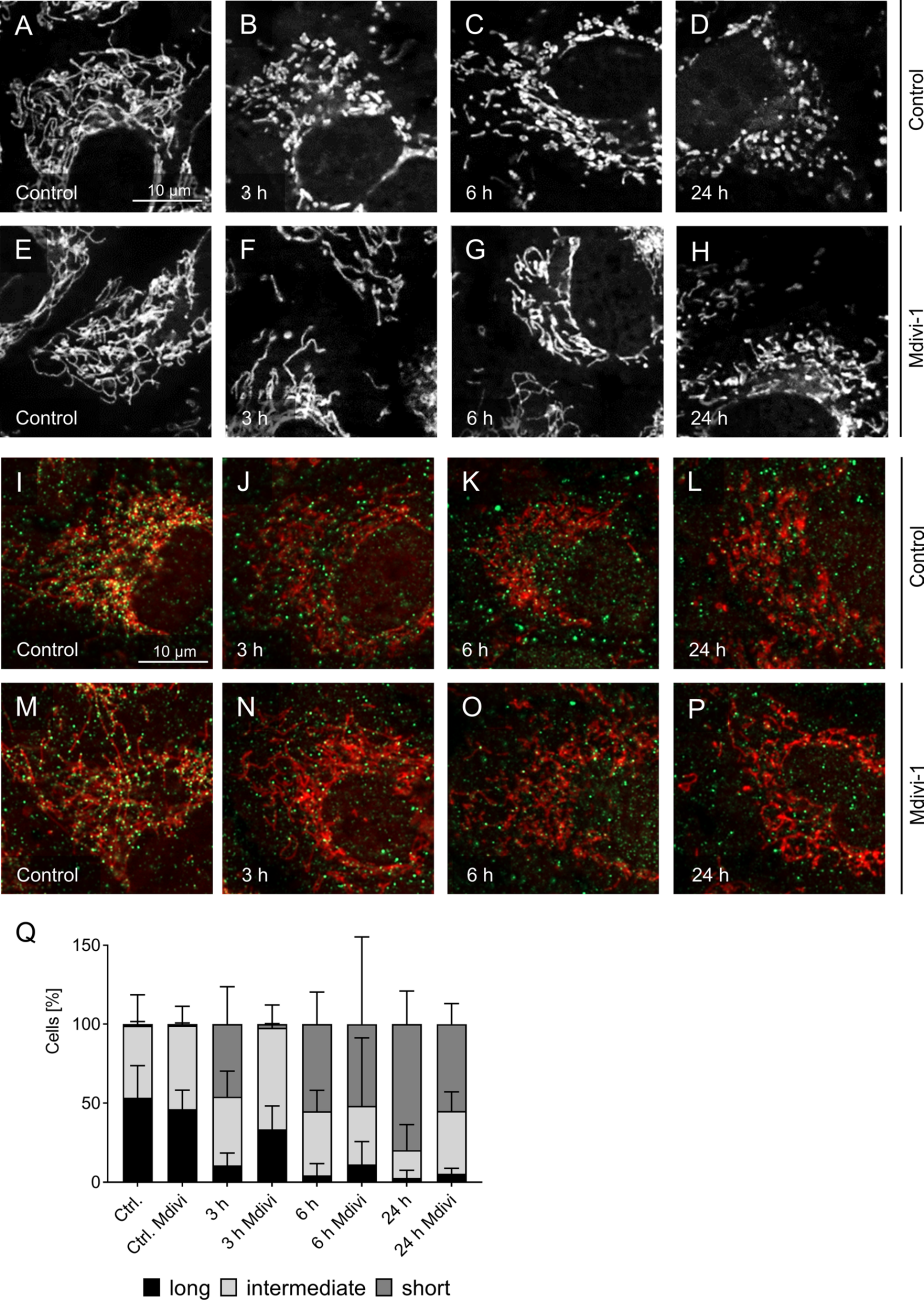
Cold-induced mitochondrial fission despite the presence of the Drp1 inhibitor Mdivi-1. Porcine aortic endothelial cells (control, **A**, **I**) were incubated at 4 °C for 3 h, 6 h and 24 h with 20 µM Mdivi-1 (**E**–**H**, **M**–**P**), a potent Drp1 inhibitor. DMSO was added as solvent control (**A**–**D**, **I**–**L**). Mitochondria were stained with MitoTracker Red. Additionally, Drp1 immunofluorescence (green) staining was applied (**I**–**P**). The images were documented by fluorescence microscopy (MitoTracker Red: λ_exc._ = 546 ± 6 nm, λ_em._ ≥ 590 nm; FITC: λ_exc._ = 470 ± 20 nm, λ_em._ = 525 ± 25 nm). Representative figures of n = 4 experiments. For quantification of mitochondrial fission (**Q**) cells were categorised in cells with predominantly short, intermediate and long mitochondria by a person blinded to the experiment. Means ± SD of n = 4 experiments

Addition of the Drp1 inhibitor Mdivi-1, although used at the high concentration of 20 µM and with pre-treatment of the cells at 37 °C, did not prevent cold-induced mitochondrial fission (Fig. 8) but only slightly delayed it. The clearest difference occurred after 3 h (Fig. 8F vs. B, Fig. 8Q); after 6 h and even more so after 24 h fission was clearly visible under both conditions with little difference (Fig. 8G vs. C, Fig. 8H vs. D, Fig. 8Q). Mdivi-1 did also not change the behaviour of Drp1 dislocating from the mitochondria during cold incubation (less co-localization; Fig. 8J–L and N–P).

### Effects of cold incubation on Opa1 processing

**Fig. 9:**
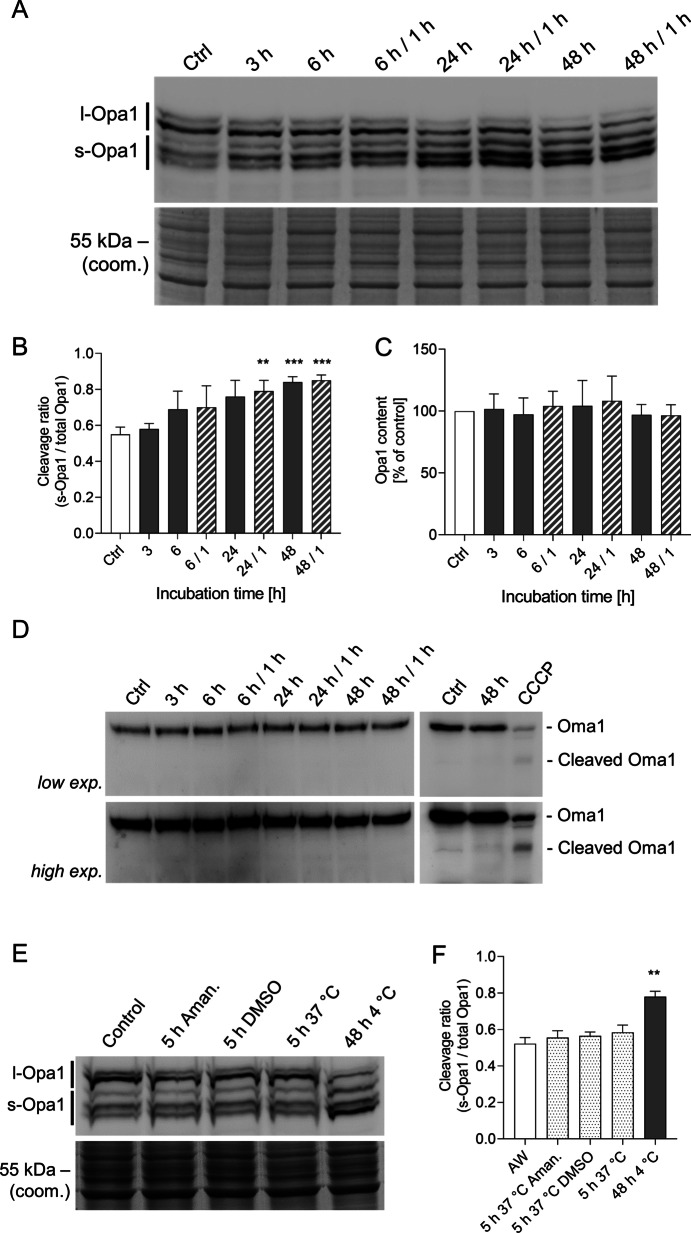
Qualitative and quantitative analysis of Opa1 and Oma1 protein levels in cells exposed to hypothermia. Protein lysates of porcine aortic endothelial cells (Control, Ctrl) incubated for 3 h, 6 h, 24 h or 48 h at 4 °C, part of them rewarmed for 1 h at 37 °C (e.g. 48/1) were analysed by western blot with antibodies against Opa1 and Oma1. The two heavier bands represent the long isoforms of Opa1 (l-Opa1) known to be involved in inner membrane fusion and the three lighter bands represent the short isoforms (s-Opa1) incapable of mitochondrial fusion (**A**, **E**). The transcription inhibitor amanitine (Aman.;10 µg/ml) was added to assess a potential shift to s-Opa1 by missing transcription and thus missing synthesis of l-Opa1 (**E**, **F**). Cleavage of Oma1 was analysed (**D**), high exposure to carbonyl cyanide *m*-chlorophenyl hydrazone (CCCP; 20 µM) served as positive control. Representative figures of n = 4 experiments. Coomassie staining was used as loading control (coom.). The band intensity was quantified using the BioVision software and plotted as s-Opa1 to total Opa1 (**B**, **F**) or total Opa1 content (**C**). Means ± SD of n = 4 experiments. Cold conditions are shown as grey bars, warm conditions are shown as open, dotted or hatched bars. ** Significantly different to control (p ≤ 0.01) *** (p ≤ 0.005)

Opa1, the central protein for inner membrane fusion and generally regarded as a major player of mitochondrial fusion, appears in five isoforms. The short isoforms (s-Opa1) result from cleavage of the long isoforms (l-Opa1) and are, in contrast to the long isoforms, not able to mediate fusion. Under normothermic control conditions, both, the long and the short isoforms of Opa1 could be observed (Fig. 9A), with the short forms accounting for about 55% of total Opa1 (Fig. 9B). Over the duration of cold incubation, there was no change in the total protein level of Opa1 (Fig. 9C), but in the ratio of s-Opa1 to total Opa1 (s-Opa1 plus l-Opa1; Fig. 9B). Only a very slight shift towards the short isoforms was visible after 3 h in the cold, i.e. at a time point when fission was already marked. Enhanced cleavage of l-Opa1 to s-Opa1 became more pronounced after longer periods of cold incubation with a marked difference at 24 h when > 70% of Opa1 was processed to s-Opa1. The short isoforms finally exceeded 80% of total Opa1 after 48 h of cold incubation. In rewarmed cells the protein levels of Opa1 isoforms were hardly altered compared to the respective cold incubation. Oma1, a major Opa1 regulator cleaving Opa1, is activated by self-cleavage. Oma1 cleavage could not be observed during cold incubation or rewarming (Fig. 9D), not even after high exposure. Controls in which Oma1 self-cleavage was induced by adding the uncoupler carbonyl cyanide *m*-chlorophenyl hydrazine (CCCP, 20 µM), in contrast, clearly showed cleaved Oma1. In order to exclude that (low) constitutive Opa1 processing, together with lacking re-synthesis of l-Opa1 in the cold, could lead to the alteration of the cleavage ratio, the transcription inhibitor α-amanitin was added to warm control incubations (for 5 h at 37 °C, a time period that should yield—with regard to the Q_10_ rule—largely equivalent metabolism as 48 h cold incubation). There were no differences between warm incubation with or without amanitin (Fig. 9E, F), i.e. inhibition of re-synthesis of l-Opa1 per se cannot explain the altered cleavage ratio after cold incubation.

### Effects of cold incubation on cellular ATP levels

**Fig. 10:**
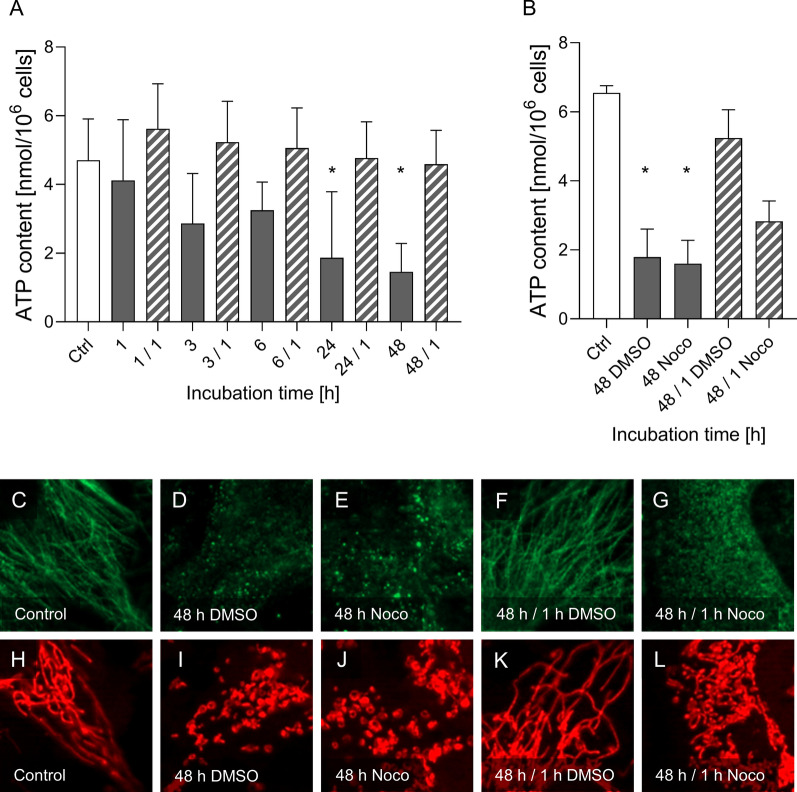
Cellular ATP content during cold incubation and rewarming. **A** Aortic endothelial cells (control, ctrl, open bar) were exposed to 4 °C for 1 h, 3 h, 6 h, 24 h and 48 h (grey bars) and rewarmed for 1 h at 37 °C (hatched bars; e.g. 48/1). **B** Additionally, ATP content was measured after 48 h at 4 °C and rewarming for 1 h at 37 °C with and without the tubulin polymerization inhibitor nocodazole (Noco; added 18 h before rewarming). ATP was measured in cell lysates with a bioluminescence assay kit. Means ± SD of n = 4 experiments. Images of the microtubuli (green; **C**–**G**) and mitochondria (red; **H**–**L**) are shown (MitoTracker Red: λ_exc._ = 546 ± 6 nm, λ_em._ ≥ 590 nm; FITC: λ_exc._ = 470 ± 20 nm, λ_em._ = 525 ± 25 nm). Representative figures of n = 4 experiments. * Significant difference to the control (p ≤ 0.05)

To assess mitochondrial function during/after cold storage cellular ATP content was measured. Under normothermic control conditions endothelial ATP content amounted to 4.7 ± 1.0 nmol ATP/10^6^ cells (Fig. 10). Over the period of cold incubation cellular ATP content gradually declined with a significant drop to 1.9 ± 1.7 nmol/10^6^ cells after 24 h and 1.5 ± 0.7 nmol/10^6^ cells after 48 h at 4 °C. During rewarming after each cold incubation period the ATP content increased again, reaching the level of the warm control within 1 h. To provide evidence that fission and fusion are important for the ATP decline during hypothermia and the ATP increase after rewarming, respectively, we performed controls in which re-fusion of mitochondria was prevented. To this end we inhibited tubulin polymerization by the addition of nocodazole (5 µM; 18 h before the end of cold incubation, after mitochondrial fragmentation occurred). Intact microtubuli could be observed in the control (Fig. 10C) and after rewarming (Fig. 10F), lack of microtubuli (depolymerized tubulin) in the cold (Fig. 10D, E) and after rewarming with nocodazole (Fig. 10G). Re-fusion during rewarming was thus inhibited and mitochondria were noticeably shorter after rewarming with nocodazole (Fig. 10L, compare to Fig. 10K). In the incubation with nocodazole the ATP content did not increase to the level of the corresponding rewarming control (solvent control) after 1 h rewarming at 37 °C and remained below 50% of the control (Fig. 10B) suggesting that fusion is important for ATP generation.

## Discussion

In this study we showed that cold-induced mitochondrial fission occurred already after short exposure to hypothermia (3 h, 4 °C) and over a wide temperature range (≤ 15 °C), that it is accompanied by unusual morphologies like donuts, that it does not appear to be clearly mediated by Drp1 and its receptors and that it appears functionally relevant.

Cold-induced mitochondrial fission occurring in isolated cells, as described here, has to be clearly differentiated to cold-induced mitochondrial fission in brown adipose tissue of whole animals; both are mechanistically totally different. Cold-induced mitochondrial fission in brown adipose tissue has been described to be triggered by the release of the stress hormone norepinephrine by the adrenal gland that is then acting on plasma membrane receptors (Wikstrom et al. 2014). Cold-induced mitochondrial fission in isolated cells or isolated organs cannot be mediated by this mechanism but must rather be triggered intrinsically in the cold-exposed cells.

Cold-induced fission in endothelial cells, i.e. the cell type that is most severely affected by hypothermia/organ preservation (McKeown et al. 1988; Rauen et al. 1994a, 2004), appeared to occur over a wide temperature range (Fig. 3). For transplantation, many different temperatures from subnormothermic temperature to supercooling are discussed for different organ preservation approaches (Bellini et al. 2019; de Vries et al. 2019; Fuller et al. 2013; Gilbo et al. 2017) and cold-induced mitochondrial fission might be relevant for most of them. The early occurrence of the enhanced fission at 4 °C (Fig. 2) underlines a potential relevance during organ preservation with many organs being stored for hours until transplantation (Jing et al. 2018). The cold-induced mitochondrial fragmentation observed here was very marked (Figs. 1, 2, 3), similar to other models of cold exposure of isolated cells (Hendriks et al. 2017; Kerkweg et al. 2003; Rauen et al. 2006; Zhang et al. 2010), but exceeding the fission described in many normothermic models by far (Barsoum et al. 2006, Toyama et al. 2016).

Mitochondrial fission as occurring under physiological and most pathophysiological conditions, i.e. under conditions of normothermia, is regarded to be mediated by the GTPase Drp1 (Kraus et al. 2021) and its receptors fission 1 protein (Fis1), mitochondrial fission factor (Mff), mitochondrial dynamics proteins of 49 kDa (MiD49) and of 51 kDa (MiD51; Pernas et al. 2016). Drp1 is then believed to oligomerize at the outer mitochondrial membrane and to initiate mitochondrial abscission by GTP hydrolysis (Pernas et al. 2016). Drp1 is known to be regulated by different posttranslational modifications such as ubiquitylation, S-nitrosylation and sumoylation, but the best established and likely most important is phosphorylation. Phosphorylation at S616 by cyclin B kinase activates fission while phosphorylation at S637 by cyclic AMP-dependent protein kinase A inhibits the GTPase activity and thus inactivates the protein (Kraus et al. 2021).

For cold-induced mitochondrial fragmentation, however, we could not find convincing evidence for this classical mechanism: Although there was phosphorylation at the activating site S616, significant phosphorylation was only observed after ≥ 24 h (Fig. 6) while most fission processes occurred within the first few hours of cold incubation leading to marked mitochondrial fragmentation already after 3 h of cold incubation (Fig. 2). Furthermore, both the activating (S616) and the inactivating (S637) site were phosphorylated in parallel (Fig. 6). Mff was also phosphorylated in the cold, while the total level of Mff did not change. And while it is described that phosphorylated Mff recruits Drp1 to the mitochondria (Zhou et al. 2018) and Mff-dependent fission can lead to apoptosis of endothelial cells (Zhou et al. 2017), we did neither observe increased Drp1 recruitment in the cold (Fig. 7) nor cell injury or apoptosis (Fig. 1J, K). The parallel phosphorylation of Mff and of Drp1 at both phosphorylation sites might suggest that the phosphorylation is either a stress response or that kinases are less cold-sensitive (i.e. have higher residual activity in the cold) than phosphatases. In all cases, strong phosphorylation occurred only after prolonged cold incubation, when most of the fission had already occurred (Fig. 6B–D). And most importantly, immunofluorescence did not show increased mitochondrial Drp1 localization during hypothermia but rather less mitochondrial Drp1 localization than under warm control conditions (Fig. 7). In addition, the small molecule Drp1 inhibitor Mdivi-1, known to be very effective (Smith et al. 2017) and targeting the GTPase activity of Drp1 (Cassidy-Stone et al. 2008) did, in line with our results on Drp1 localization (Fig. 7), only slightly delay cold-induced mitochondrial fission but not prevent it (despite pre-incubation and high concentration; Fig. 8A–H, Q). Furthermore it did not alter Drp1 localization (Fig. 8I–P). Together, this could mean that cold-induced mitochondrial fission is partially or largely Drp1-independent—in contrast to mitochondrial fission in most models (Kraus et al. 2021; Smirnova et al. 2001).

Not only enhanced fission but also reduced fusion could lead to fragmented mitochondria at 4 °C. The central enzyme for fusion of the inner mitochondrial membrane Opa1 exists in different isoforms: Three short isoforms (s-Opa1) involved in the maintenance of cristae structure, and two long isoforms (l-Opa1) required for fusion (Lee et al. 2017). Opa1 is highly regulated by differential splicing and proteolytic processing by Yme1l or the stress-induced metalloprotease Oma1 (Pernas et al. 2016), which itself has been described to get activated by uncoupling and depolarisation of mitochondria (Baker et al. 2014; Jones et al. 2017; Toyama et al. 2016; Zhang et al. 2014). However, marked Opa1 cleavage only occurred after ≥ 6 h of cold incubation (Fig. 9A, B), while marked mitochondrial fragmentation was already present after 3 h of cold incubation (Fig. 2). In addition, even after 48 h of cold incubation mitochondria were still fusion-competent despite the decreased levels of l-Opa1 (Fig. 9) as rapid re-fusion after rewarming demonstrated (Fig. 1). This—in conjunction with the fact that reduced fusion would not lead to mitochondrial fragmentation without fission occurring—renders the loss of l-Opa1 unlikely to be the central mechanism responsible for cold-induced mitochondrial fragmentation. Nevertheless, the shift in the Opa1 cleavage ratio was marked after 48 h. It appeared to be due to true processing rather than decreased expression of full-length Opa1 (Fig. 9E, F). However, Oma1 protein levels did not change during cold incubation and no activation by cleavage was detectable (Fig. 9D).

In the literature cold-induced mitochondrial fragmentation was only addressed mechanistically in a few studies. In models of cold ischemia of the kidney, i.e. in models where renal cells were subjected to both, hypothermia and hypoxia, Parajuli et al. (2017) described, for the rat kidney, a loss of diverse fission and fusion proteins, but samples were only taken 24 h after reperfusion, i.e. long after rewarming. For the mouse kidney, Zhu et al. (2020) described PKCδ-dependent phosphorylation of Drp1 at S616, but did not provide data on S637. In HEK-293 cells exposed to hypothermia in DMEM cell culture medium (without CO_2_), Hendriks et al. (Hendriks et al. 2017) observed increased mitochondrial expression of Drp1 and Zhang et al. (2010) observed fluctuating levels of Drp1 and of Mfn2, findings that we did not see in endothelial cells. In addition Zhang et al. showed that silencing of Mfn2 increased injury, suggesting that re-fusion is important for cellular survival. Whether Drp1 is crucial for mitochondrial fission also under hypothermic conditions, was not addressed in either of these studies. Most interestingly, in plants cold-induced mitochondrial fission was also observed when *Arabidopsis thaliana* was incubated at 4 °C and described to follow a different fission pathway than under normothermic conditions (Arimura et al. 2017).

In addition to fragmented, i.e. short or very short (dot-like) mitochondria, we also observed unusual mitochondrial morphologies under cold conditions in this study, namely donuts, lassos and blobs (Fig. 4). Donuts, i.e. short mitochondria auto-fused end-to-end (Fig. 4E, F), have also been observed under hypoxia and were regarded as a protection mechanism against volume alterations (Liu et al. 2011). Lasso-like structures occur when mitochondria auto-fuse end-to-side. Blobs have been described to be swollen donuts (Ahmad et al. 2013), but, without time-lapse images or electron microscopy cannot be safely distinguished from short, swollen mitochondria. Donut and blob formation have been shown to be related to perturbations of mitochondrial Ca^2+^ and mitochondrial ROS formation in lung cells (Ahmad et al. 2013). Most notably, as donut and lasso formation require auto-fusion, their occurrence in our study is evidence that fusion is possible in hypothermia.

While there was no indication for a role of cell injury or apoptosis for cold-induced mitochondrial fission during cold incubation (Fig. 1G–K), it is not clear whether cold-induced mitochondrial fission is a physiological adaptation to the low cellular energy demand during hypothermia or whether it is an expression of stress. Interestingly, hamster kidney cells, i.e. cells from a hibernating animal, showed already short mitochondria under warm conditions (Hendriks et al. 2017). On the contrary the finding that ATP levels severely decreased during cold incubation (Fig. 10A) suggests that the mitochondrial fragmentation is not a mere adaptation to decreased energy demand but rather a reason for or a consequence of decreased ATP formation. The finding that the prevention of re-fusion after rewarming leads to reduced ATP production (Fig. 10B) could be an indication that fission is causing the low ATP content.

In our experiments, i.e. after cold aerobic incubation in a solution of physiological extracellular ion composition, cold incubation for ≤ 48 h and in the presence of the iron chelator deferoxamine which prevented cold-induced cell injury completely in these cells (Fig. 1G–K; in line with Rauen et al. 2004), cold-induced mitochondrial fission was completely reversible during rewarming and mitochondrial function was normalized. However, there is evidence that re-fusion is hampered or completely lost and mitochondrial and/or cellular injury occurs upon rewarming/reperfusion in the absence of an iron chelator (Kerkweg et al. 2003), after prolonged cold incubation (Pless et al. 2012), in other solutions (Klüsener et al., unpublished result) and under conditions of combined hypoxia and hypothermia (Parajuli et al. 2017, Zhu et al. 2020). Whether mitochondrial fission leads to the ATP decline (Pernas et al. 2016) under these conditions or whether additional functional changes contribute is not known yet.

We here showed that cold-induced mitochondrial fission occurs early, over a wide temperature range, by an unusual, at least partially Drp1-independent mechanism and has functional consequences. With regard to the scarce data on cold-induced mitochondrial fission we decided to concentrate on the pure model of hypothermic exposure uncomplicated by further injurious factors to characterize cold-induced mitochondrial fission. To delineate the interplay with hypoxia and the impact of preservation solutions in diverse transplantation-relevant cell types/tissues will be the logical next step. As mitochondrial energy generation is a prerequisite for both organ regeneration and organ function, understanding the disturbances of mitochondrial dynamics in the long run likely offers opportunities for improvements in organ preservation and particularly in the newly arising field of organ reconditioning.

## Supplementary Information

**Additional file 1:** Video file of live cell imaging of stained mitochondria of porcine aortic endothelial cells. The cells were stained with MitoTracker Red and incubated at 8 °C for 5:40 h (18 images to minimize phototoxicity).

**Additional file 2:** Video file of live cell imaging of stained mitochondria of porcine aortic endothelial cells slowly rewarmed after 48 h at 4 °C to 37 °C. The cells were stained with MitoTracker Red (18 images to minimize phototoxicity).

## Data Availability

The datasets generated and analysed during the current study are available from the corresponding author on reasonable request.

## Abbreviations

DMSO: Dimethyl sulfoxide
Drp1: Dynamin-related protein 1
Fis1: Mitochondrial fission 1 protein
FITC: Fluorescein isothiocyanate
HBSS: Hanks' Balanced Salt Solution
IgG: Immunoglobulin G
KH: Krebs–Henseleit
LDH: Lactate dehydrogenase
M199: Medium 199
Mdivi-1: Mitochondrial division inhibitor 1
Mff: Mitochondrial fission factor
Mfn: Mitofusin
MiD: Mitochondrial dynamics protein
Opa1: Optic atrophy 1
Oma1: Overlapping proteolytic activity with m-AAA protease 1
ROS: Reactive oxygen species
RT: Room temperature
TMRM: Tetramethylrhodamine methyl ester
UW: University of Wisconsin
